# Sustaining and scaling a clinic-based approach to address health-related social needs

**DOI:** 10.3389/frhs.2023.1040992

**Published:** 2023-02-17

**Authors:** MaryCatherine Arbour, Placidina Fico, Baraka Floyd, Samantha Morton, Patsy Hampton, Jennifer Murphy Sims, Sidney Atwood, Robert Sege

**Affiliations:** ^1^ Division of Global Health Equity, Department of Medicine, Brigham and Women's Hospital, Boston, MA, United States; ^2^ Department of Pediatrics, Stanford School of Medicine, Stanford, CA, United States; ^3^ MLPB, Boston, MA, United States; ^4^ Center for the Study of Social Policy, Washington, D.C., United States; ^5^ Early Intervention Services, UCSF Benioff Children's Hospital, Oakland, CA, United States; ^6^ Institute for Clinical Research and Health Policy Studies, Tufts Medical Center, Boston, MA, United States

**Keywords:** scale - up, quality improvement, pediatric primary care, social determinansts of health, well child care, interdiscipinarity approach, data driven adaptation, evidence based interventions in primary care

## Abstract

**Objective:**

Scaling evidence-based interventions (EBIs) from pilot phase remains a pressing challenge in efforts to address health-related social needs (HRSN) and improve population health. This study describes an innovative approach to sustaining and further spreading DULCE (Developmental Understanding and Legal Collaboration for Everyone), a universal EBI that supports pediatric clinics to implement the American Academy of Pediatrics' Bright Futures™ guidelines for infants' well-child visits (WCVs) and introduces a new quality measure of families' HRSN resource use.

**Methods:**

Between August 2018 and December 2019, seven teams in four communities in three states implemented DULCE: four teams that had been implementing DULCE since 2016 and three new teams. Teams received monthly data reports and individualized continuous quality improvement (CQI) coaching for six months, followed by lighter-touch support *via* quarterly group calls (peer-to-peer learning and coaching). Run charts were used to study outcome (percent of infants that received all WCVs on time) and process measures (percent of families screened for HRSN and connected to resources).

**Results:**

Integrating three new sites was associated with an initial regression of outcome: 41% of infants received all WCVs on time, followed by improvement to 48%. Process performance was sustained or improved: among 989 participating families, 84% (831) received 1-month WCVs on time; 96% (946) were screened for seven HRSN, 54% (508) had HRSN, and 87% (444) used HRSN resources.

**Conclusion:**

An innovative, lighter-touch CQI approach to a second phase of scale-up resulted in sustainment or improvements in most processes and outcomes. Outcomes-oriented CQI measures (family receipt of resources) are an important addition to more traditional process-oriented indicators.

## 1. Introduction

Evidence-based interventions (EBIs) delivered through the pediatric medical home can improve outcomes among families with young children (1). Integrating health-related social needs (HRSN) screening and support into primary care is a priority strategy to improve population health outcomes (2). The American Academy of Pediatrics (AAP) Bright Futures™ 4th Edition (BF4) recommends that pediatric clinics address HRSN during well-child visits (WCVs) (3).

Evidence for effectiveness of interventions addressing HRSN in pediatric settings comes mostly from pilot-sized studies (4). However, in the real world, many small-scale pilot studies demonstrating effectiveness are never implemented widely (5, 6). The American Academy of Pediatrics noted that most efforts to expand successful pilot interventions at scale result in disappointingly small effect sizes (7). Emerging literature describes frameworks for large-scale dissemination of EBIs, factors that facilitate uptake, and lessons learned from scale-up attempts (5, 8–13), but there is no established single best approach for scaling EBIs to improve population health (10).

The Institute for Healthcare Improvement's Breakthrough Series Collaborative model (BTS) has demonstrated potential to increase uptake of EBIs across diverse contexts (14–18), by combining continuous quality improvement methodologies and networked peer learning. Continuous quality improvement methods (CQI) engage the entire organization and its frontline providers in a series of ongoing observations, adjustments, and interventions to produce measurable improvements in outcomes (19, 20). Central to this approach are Plan-Do-Study-Act (PDSA) cycles, which allow cross-hierarchical teams of service providers to identify ideas they believe might improve outcomes; they **plan** a small test of that idea, **do** that test in real day-to-day practice, **study** how the test was executed and what resulted (using observations and data), and **act** on those results – i.e., abandoning ideas that did not work, adapting ideas that seemed promising but in need of optimization (then re-testing), or adopting into practice ideas that worked optimally in their contexts. By ensuring that change ideas are tested and adapted to the local context by frontline teams making real-time, data-based decisions, PDSA testing facilitates adaptive design that can accommodate different contexts as an intervention is scaled up (21).

**Figure 1:**
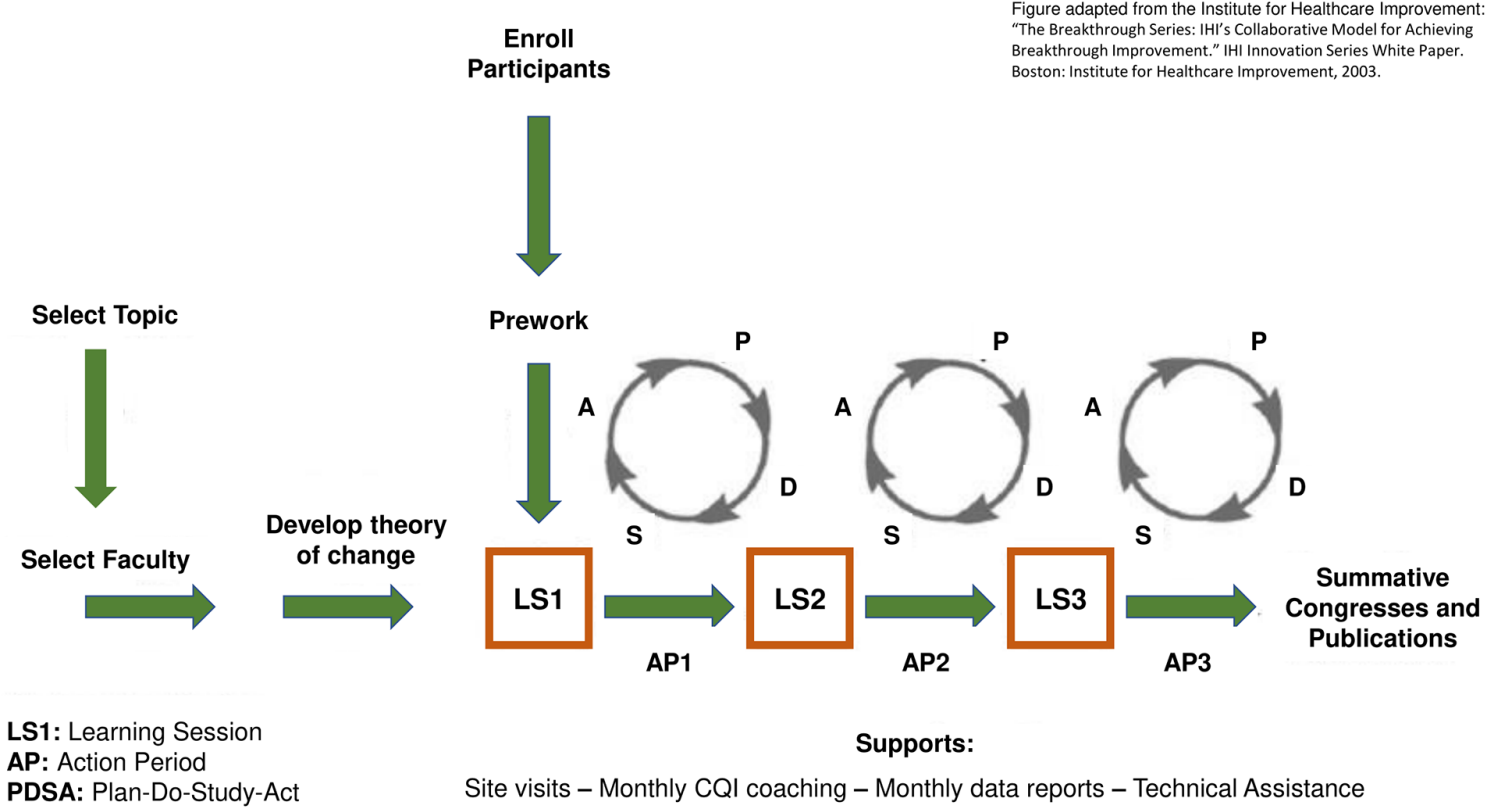
The Institute for Healthcare Improvement's Breakthrough Series Learning Collaborative.

The Breakthrough Series Collaborative model (BTS) combines CQI methodologies with networked peer learning (22). It recruits teams of direct service providers and stakeholders to pursue one shared, specific aim during a defined period of time, typically 9 to 18 months, and creates a structure wherein interested organizations can learn from each other and recognized experts. The model has three core elements: 1) learning sessions that bring teams together periodically for training and collaboration, separated by 2) “action periods” during which teams test what they have learned in practice, using 3) Plan-Do-Study-Act cycles – the structured approach for learning from rapid-cycle testing of innovations in practice. Figure 1 depicts this traditional BTS structure.

Continuous quality improvement and BTS methods have been applied widely. The CQI approach emerged in the 1950s to overcome manufacturing deficiencies (23, 24) and has subsequently been applied in healthcare, public health, nonprofit and public management and, recently, education (25–28). Since 1995, the BTS model has increased uptake of EBIs and improved outcomes in public health (20, 27, 29), including improved rates and reduced disparities in immunizations (30), spread of trauma-informed care practices in child welfare (31, 32), and improved breastfeeding, developmental promotion, and caregiver depression outcomes in home visiting programs (33–35). In clinical medicine, BTS has been applied to several dozen topics involving over 2,000 teams from 1,000 healthcare organizations to achieve concrete results: reducing waiting times by 50%, worker absenteeism by 25%, ICU costs by 25%, hospitalizations for patients with congestive heart failure by 50%, and eliminating 100,000 deaths due to medical errors (15, 36). More recently, the Breakthrough Series has entered the education field as “Networked Improvement Communities.” (37–40). For example, two attendance-focused BTS collaboratives increased school attendance: one from 44.9% to 59.2% at seven early childhood education centers in New Zealand (41); another from 83.7% to 87.1% at five public preschools in rural Chile (42).

This study reports an effort to scale DULCE (Developmental Understanding and Legal Collaboration for Everyone) – an evidence-based, cross-sector intervention for addressing HRSN among families with infants that is delivered through pediatric primary care. A first effectiveness trial of DULCE, conducted at a single site with 330 families demonstrated that DULCE increased preventive care adherence and accelerated families' access to HRSN supports (43). A subsequent study of DULCE expansion to five sites serving 692 families used a BTS Collaborative model as its scale framework and replicated these findings - increased on-time WCVs and accelerated access to HRSN resource information (44). That application of the BTS model provided resource-intensive support including four in-person, group training sessions (12 days total), two or three coaching contacts each month (including monthly group implementation webinars and individual site CQI coaching), and two site visits.

Tailoring the type and intensity of support over time as an EBI spreads is crucial, since resources (e.g., time, technical, financial) are often limited (45), and different phases of scale may require different supports (46). Extant literature suggests that later phases of expansion may require less intensive (and less costly) supports because they benefit from people who participated in early phases of expansion championing the EBI and mentoring their peers. In addition, experience gained in the earliest phase of scale – experience testing the theory of change under a broad range of conditions, developing infrastructure and human capacity to support the method being used to scale up, etc.— may facilitate acceleration in the rate of EBI adoption (8). However, sustained implementation of EBIs in real-world settings is a considerable challenge, and many fail to continue once support is decreased or removed.

This study examined whether a lighter-touch application of the same BTS scale framework could be used to sustain DULCE practice improvements in four established sites and spread practice improvements to three new clinic sites. In addition, it added new effectiveness data by measuring families' HRSN resource use. Specifically, it answers two research questions:

1. Can a lighter-touch application of BTS sustain improvements achieved during a prior expansion by four established DULCE teams and spread improvements in on-time WCVs and identification and support for HRSN to three new clinics?
2. What proportion of families use resources after receiving resource information for identified HRSN?

## 2. Materials and methods

### 2.1. Intervention

DULCE (Developmental Understanding and Legal Collaboration for Everyone) is a universal, evidence-based pediatric primary care approach for families with infants from birth through 6 months of age. DULCE embeds a community health worker (“Family specialist,” FS) within a cross-sector team that includes an early childhood system representative, legal partner, clinic administrator, and pediatric and behavioral health clinicians. The team works together to link families to needed resources.

**Figure 2:**
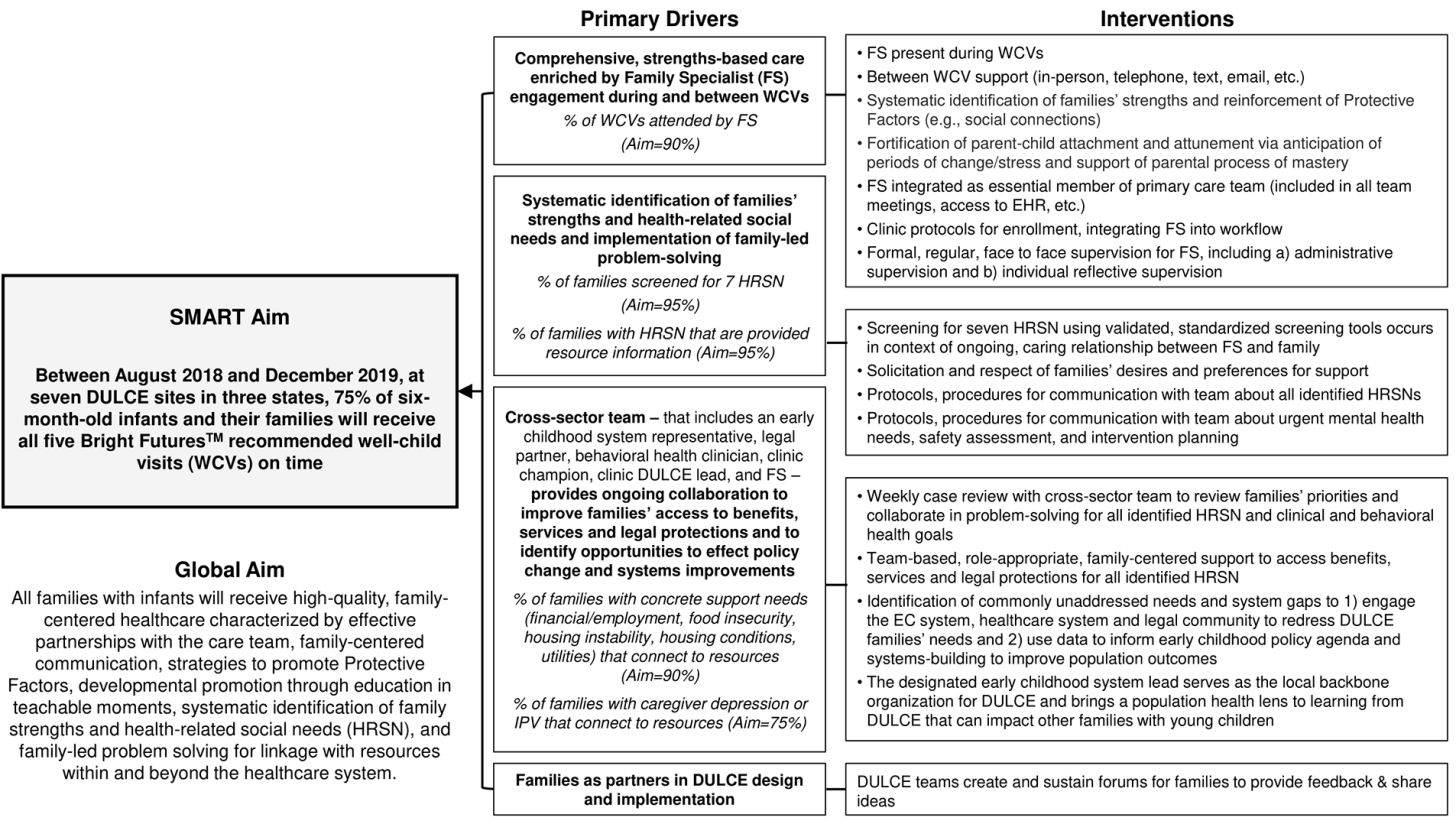
DULCE Key Driver Diagram.

DULCE's theory of change for improving completion of preventive care is visualized in a driver diagram with four primary drivers – that is, key determinants – that contribute to reaching the goal of on-time well-child visit completion (see Figure 2). The first driver focuses on comprehensive care enriched by a Family Specialist (FS, i.e., a community health worker) who attends WCVs, reinforces protective factors, offers developmental guidance and is families' most frequent point of contact. All FS received Brazelton Touchpoints^TM^ training (47). The second driver concentrates on identification of families’ strengths and HRSN and family-led problem-solving across seven evidence-based HRSN domains: caregiver depression, intimate partner violence (IPV), housing conditions, housing instability, food insecurity, employment/financial needs, and utilities.

The third driver emphasizes the cross-sector team that includes the FS, an early childhood system representative, legal partner, clinic administrator, and pediatric and behavioral health clinicians. This team conducts weekly case reviews; collaborates to support families' access to benefits, services and legal protections; and identifies opportunities to effect policy and systems improvements (48). The fourth driver prioritizes families as partners *via* diverse strategies, (e.g., *via* exit surveys, periodic celebrations with focus groups, and as DULCE CQI team members).

### 2.2. Implementation strategy

In previous work (hereafter referred to as “phase one”), a resource-intensive BTS collaborative succeeded in scaling DULCE to five clinical sites that increased on-time WCVs and accelerated access to HRSN resource information. This study (“phase two”) aimed to use a lighter-touch application of BTS to sustain improvements in four established DULCE teams (one site from phase one discontinued DULCE due to institutional leadership transitions) and spread improvements to three new clinics.

In both phases, DULCE's implementation was guided by the principles and core components of the BTS model. DULCE sites formed teams of direct service providers and stakeholders that included the Family Specialist, early childhood system representative, legal partner, clinic administrator, and pediatric and behavioral health clinicians. Teams committed to pursue one shared, specific aim (75% of 6-month-old infants and their families receive all five recommended WCVs on-time) during a defined period of time (19 months in phase one, 17 months in phase two).

In both phases, DULCE implementation was supported by the DULCE National team, comprised of staff from the lead organization, the Center for the Study of Social Policy (CSSP), together with DULCE model developers, a CQI expert, practicing pediatrician, and infant mental health specialists. CSSP staff had extensive experience and established relationships with Early Childhood Systems leaders nationally. DULCE model developers created the DULCE approach and conducted the original randomized controlled trial (RCT), then remained actively involved through phase one and phase two expansion not only as thought partners and strategists, but also as teachers, mentors and coaches to DULCE site teams and their role-specific counterparts (e.g., clinic leaders, providers, and legal partners). Similarly, the practicing pediatrician and infant mental health specialists each led portions of trainings and coached teams, clinicians, and Family Specialists. The CQI expert supported site teams in using data to inform practice and in applying CQI tools (e.g., PDSA cycles, process maps) to adapt DULCE interventions to work well in their local contexts.

In both phases, efforts were made to include DULCE team members who shared lived experience with the DULCE families. This involved hiring Family Specialists from similar racial, ethnic and linguistic backgrounds to the patients served by DULCE. In practice, this meant hiring Family Specialists with bachelor's or paraprofessional educational level, more consistent with a community health worker profile, in contrast with the original RCT that used masters-level Family Specialists. The decision to change the profile of the FS job description reflected two priorities: an effort to accommodate different cultures and languages to better serve local populations, as well as an effort to design for sustainability and scale, since requiring masters-level FS might limit DULCE's potential reach.

Like Family Specialists, DULCE clinics and teams reflect the populations they serve, often coming from the same communities as families. DULCE team members are culturally and linguistically equipped to care for their local populations, which maximizes the impact of their various areas of expertise (e.g., legal, behavioral health). Furthermore, DULCE print and multimedia materials are available in both English and Spanish. When a family cannot communicate in any of the languages spoken by their care team, clinic interpreters are available. DULCE teams also took advantage of trainings that were offered by cross-sector partners for their own employees in order to further develop their teams' capacities to serve their communities. For example, early childhood systems invited DULCE Family Specialists and behavioral health clinicians to participate in trainings on empathic inquiry, cultural humility, and other topics originally designed for public health home visitors. Legal partners invited clinical staff and early childhood partners to attend educational *charlas* they provided for immigrants and other patients on “Know Your Rights.”.

The DULCE National team created a structure wherein teams learned from each other and DULCE National using in-person learning sessions, virtual webinars, and individual team coaching. In addition, teams exchanged learning, identified gaps in process and outcome performance, and drafted PDSA cycles to test and adapt DULCE's intervention elements between Learning Network Calls, until they worked effectively in their own context. Throughout, teams shared data, lessons, and best practices to improve collectively.

**Figure 3:**
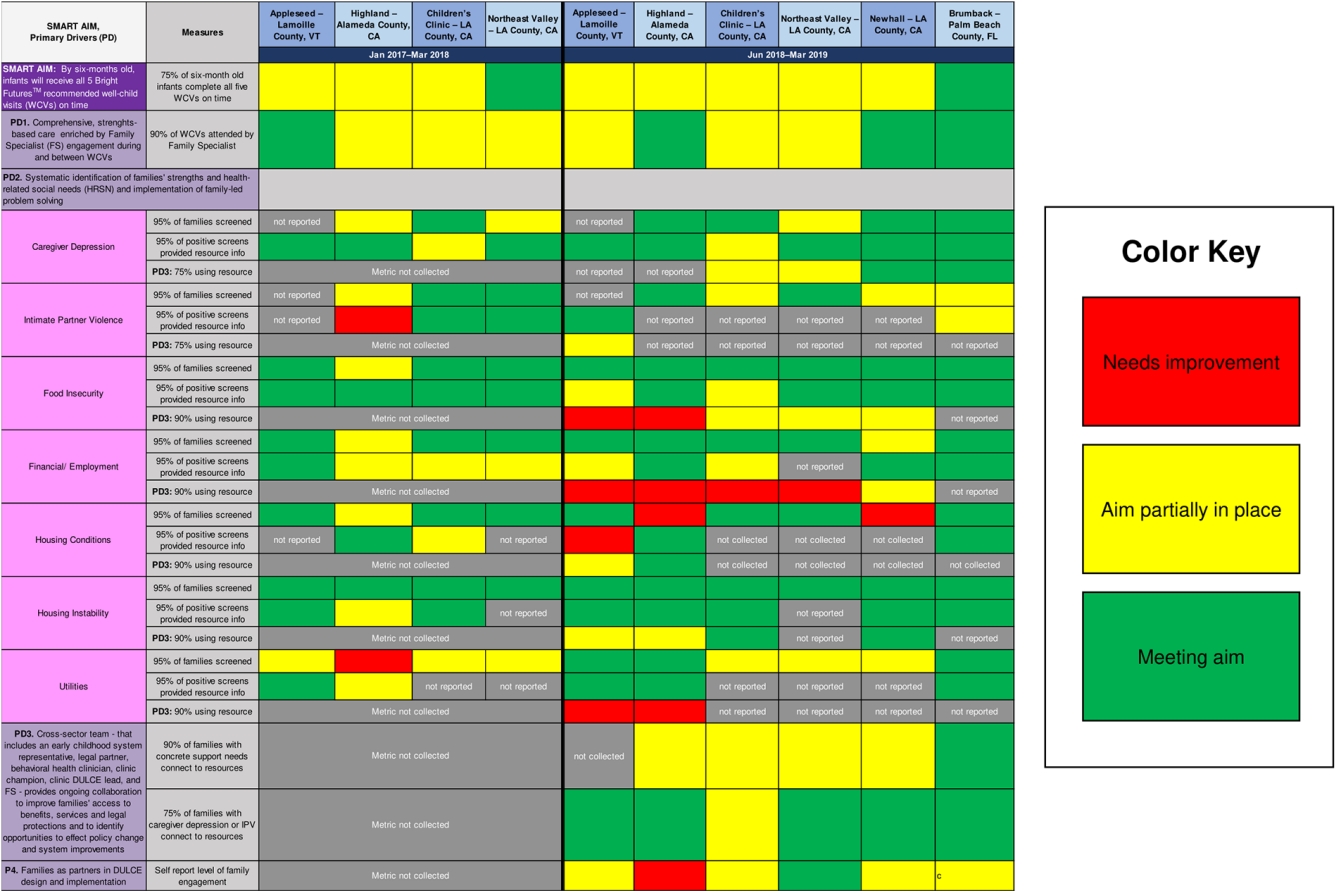
DULCE's balanced scorecard, which reflects its Key Driver Diagram measures, assessed sites’ implementation strengths and priority areas for improvement during two periods: January 2017–march 2018 (phase one; established sites only) and June 2018–march 2019 (phase two; new and established sites). These were self-assessments, and some sites did not report certain measures.

DULCE teams' readiness and capacity for integrating DULCE practices and adapting them using CQI methods varied, which is expected and desirable within the BTS model. Rather than controlling for differences in organizational capacities, DULCE National facilitated activities at Learning Sessions for local site teams to identify gaps in process and outcome performance, then summarized teams' performance using a Balanced Scorecard (Figure 3). In April 2018 (four months prior to the start of this study period) and again in April 2019, sites compared their DULCE data (not inclusive of April data) against the Key Driver Diagram's aims, assessing how well they were implementing DULCE processes and meeting DULCE outcomes (needs improvement, partially in place, meeting aim). The Balanced Scorecard identified for DULCE National focused teaching topics and local site teams with strong performance in those areas who could teach alongside DULCE National to demonstrate how they successfully implemented that specific DULCE practice. Simultaneously, the Balanced Scorecard made it easy for each team to talk about their gaps and select priority areas for PDSA testing.

New and established DULCE teams designed PDSA cycles to address implementation challenges and drafted process maps with action plans for testing. Teams initiated PDSA cycles by generating predictions about how certain change ideas would impact implementation; then **plan** how to enact the change idea; **do** what they planned; **study** the data collected; and, finally, **act** on how to move forward with the change to achieve the desired results (adopt, adapt, abandon). Supplement 1 shows the PDSA form teams used to guide this process. In keeping with best practices, DULCE teams held monthly CQI meetings where they reviewed PDSAs and solicited feedback from all DULCE team members and clinic staff involved in testing the change idea, as well as DULCE families (e.g., exit surveys).

**Figure 4:**
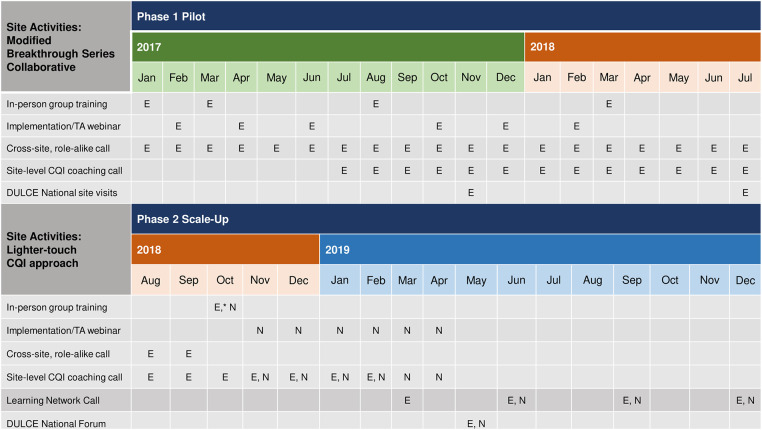
Comparison of CQI support during phases 1 & 2 of DULCE scale-up: established sites (“E”) and new sites (“N”) underwent different levels of CQI intensity when first implementing DULCE.

Figure 4 provides a comparison of support to sites during phases one and two. In phase one, between January 2017 and July 2018, the resource-intensive BTS support included four (4) two- or three-day, in-person Learning Sessions for all DULCE team members from all sites with the DULCE National team; bi-monthly group implementation webinars (total = 6); cross-site, role-alike calls (e.g., Family Specialists from all sites together (18 calls), legal partners from all sites together (12 calls), Early Childhood Leads all together [(18 calls), providers and clinic administrators all together (3 calls); (total = 51)]; monthly site-level CQI coaching with DULCE National's CQI Lead and data reports provided by DULCE National (13 per site = 52 total); and two site visits per site from the DULCE National team (8 total). Phase two's lighter-touch BTS support included two in-person convenings: an initial two-day in-person training where DULCE National provided training about DULCE and CQI methods and established DULCE teams presented illustrative examples (e.g., a role-play of cross-sector case review), and a second all-site convening halfway through (DULCE National Forum) with expert speakers and team presentations; monthly implementation webinars for new sites (6 total) followed by quarterly all-site webinars (4 total), two Family Specialist role-alike calls, monthly individual-level site-level CQI coaching with data compiled by the sites (6 per site = 42 total), and no site visits.

Thus, the phase two teams received fewer in-person trainings (2 v. 4) and site visits from the DULCE National team (0 v. 2) and fewer virtual supports (2 v. 51 role-alike group calls) for shorter duration (monthly cross-site implementation webinars for 6 months, followed by quarterly v. bimonthly webinars for 12 months; monthly CQI coaching for 6 months v. 13 months). Furthermore, phase two teams managed their own data locally, whereas in phase one, DULCE National managed all data in a centralized registry and provided monthly site-level data reports that included process and outcome measures.

### 2.3. Intervention study

To recruit participants for DULCE spread, the Center for the Study of Social Policy contacted its Early Childhood Learning and Innovation Network for Communities (EC-LINC), a national network of 14 communities that are early childhood systems innovators. In the first phase of expansion, three communities volunteered and recruited clinics serving predominantly Medicaid-insured patients and local public interest law organizations to form DULCE teams. In this second phase, the Los Angeles community added two additional clinics from healthcare systems that installed DULCE in phase one (Table 1). A third DULCE site, Palm Beach County, joined this CQI cohort during phase two; they began implementing DULCE during phase one but did not collect data nor participate in CQI until phase two and are thus considered part of this new cohort.

**Table 1:** Cross-sector DULCE team members and participating communities.

	Early Childhood System Lead Agencies	Clinic Partners	Legal Partners
Unique contribution	Accountable for a local system of services for families with young children	Offer universal reach and longitudinal relationships with families	Offer a professional orientation toward problem-solving and advocacy
Expertise	Well-versed in community resources for families and training opportunities for FS	Well-versed in the use of standard protocols to improve quality of care	Well-versed in family rights and system responsibilities
Role on team	Inform team of available community resources, champion evidence-informed practices, influence policy	Provide ongoing monitoring of families’ status and coaching of the FS to respond to unique infant and family circumstances	Lend a policy lens and expertise, offer ongoing identification of supports and strategies to address family needs
Communities^a^			
Alameda County, CA	First 5 Alameda County	Highland Pediatric Clinic (Oakland, CA)	East Bay Community Law Center
Lamoille Valley, VT	Lamoille Family Center	Appleseed Pediatrics	Vermont Legal Aid
Palm Beach County, FL	Children's Services Council of Palm Beach County	**C.L. Brumback Health Center**	Legal Aid Society of Palm Beach County, Inc.
Los Angeles County, CA	First 5 Los Angeles	The Children's Clinic (Long Beach, CA)	Legal Aid Foundation of Los Angeles
		**The Children's Clinic – The S. Mark Taper Foundation Health Center**	
		Northeast Valley Health Corporation, Sun Valley	
		**Northeast Valley Health Corporation, Newhall Health Center**	

^a^ New DULCE clinical sites that participated in the second phase of scale-up have been bolded.

#### 2.3.1. Data and measures

Family Specialists entered individual-level data into an online, custom-built registry. Demographic characteristics included infant sex; caregiver role, age, marital status, race, and ethnicity; household composition (number of adults and children) and language(s) spoken in the home. Implementation measures included the proportion of families offered participation that enrolled in DULCE, the proportion of enrolled families that completed DULCE, duration of participation (in weeks), number of encounters, and total contact time between families and the Family Specialist. A measure of case review implementation was added for phase two (percent of families discussed in case review at least once within 2 months of DULCE enrollment) because experience from phase one taught us that weekly cross-sector case review was difficult for teams to implement, initially. It was new and logistically challenging to get all DULCE team members in the same room at the same time, especially during clinic operating hours when patient care is the priority. However, once teams experienced case review, it was highly valued and became self-sustaining. As one team leader shared at the October 2018 onboarding when established teams were asked to provide a piece of advice for new DULCE teams: “Commit yourself to weekly cross-sector case review; it's the heartbeat of DULCE.”.

Process measures aligned with the primary drivers: PD1) percent of WCVs attended by the FS, PD2) percent of families that were screened for seven HRSN using validated, standardized screening questions, and, among positive screens, the percent of families provided resource information PD3) percent of families with identified HRSN that used HRSN resources.

The main outcome was the percent of six-month-old infants who received all recommended WCVs on time. It includes infants who completed the intervention and received five WCVs on time and infants who dropped out and received all recommended WCVs on time up to the date of dropout. DULCE National defined “on time” based on precedent (44, 49).

Families' data was aggregated to the clinic site where they received care, except for the Children's Clinic–Los Angeles (LA) sites [Central Long Beach Family Health Center, S. Mark Taper Foundation Health Center (SMTF)], whose data was reported together. As a result, there are data from one mixed new-established site (Children's Clinic–LA), two new sites (Newhall–LA and Brumback–Palm Beach), and three established sites (Appleseed–Lamoille, Highland–Alameda, Northeast Valley–LA).

### 2.4. Definition of the sample

Families with newborns up to 8 weeks of age were enrolled at their first office visit, excluding newborns hospitalized for >7 days after birth because they may warrant specialized services. At sites with more newborns than one FS could serve, DULCE was offered to a randomly selected subset. Clinics introduced DULCE as part of routine care, included information about DULCE in welcome packets, and introduced the FS as a care team member at the first WCV. Families could opt out.

Newborn enrollment (up to 8 weeks of age) was ongoing and continued beyond the study period. This report includes babies born June 2018 through December 2019 and followed through their six-month WCV.

Descriptive statistics were calculated for patient demographic characteristics by site and for the complete analytic sample, which includes 989 families with infants born June 2018 through December 2019 (Table 2). To describe the reach of phase two expansion, Table 3 presents a comparison of the early childhood system's catchment population (i.e., the county) to DULCE-enrolled families (the “County” and “DULCE” columns, respectively). To examine how well-aligned DULCE team members' and DULCE families’ backgrounds were, the “Team” column of Table 3 presents the composition of each site's DULCE team, which largely reflected the racial, ethnic, and linguistic makeups of their communities and/or DULCE families. Besides the DULCE site in Vermont, which enrolled 127 of 346 newborns (37%) in the county, all other sites enrolled less than 1% of infants in the early childhood system's catchment area. To put in perspective how many families DULCE reached within each site's healthcare system, we also summarized the number of infants enrolled relative to the system-level and clinic-level newborn populations (Table 4). Similarly, the Vermont site reached a higher proportion of its system-level and clinic-level infants (49.0% and 89.4%, respectively), compared to the California sites' system-level and clinic-level reach, which ranged 8.3%–12.5% and 14.3%–50.0%, respectively.

**Table 2:** Demographic characteristics of DULCE families by site.

	Total	Appleseed – Lamoille County, VT	Highland – Alameda County, CA	Northeast Valley – LA County, CA	Children's Clinic^a^ – LA County, CA	Newhall^b^ – LA County, CA	Brumback – Palm Beach County, FL
	*N* (%)	*N* (%)	*N* (%)	*N* (%)	*N* (%)	*N* (%)	*N* (%)
Full sample	989 (100)	127 (100)	194 (100)	146 (100)	249 (100)	155 (100)	118 (100)
Child sex^c^							
Male	523 (52.9)	60 (47.2)	111 (57.2)	70 (47.9)	131 (52.6)	93 (60.0)	58 (49.6)
Female	465 (47.1)	67 (52.8)	83 (42.8)	76 (52.1)	118 (47.4)	62 (40.0)	59 (50.4)
Primary caregiver^d^							
Mother	974 (98.9)	124 (98.4)	192 (99.0)	143 (99.3)	246 (98.8)	151 (98.1)	118 (100)
Father	3 (0.3)	1 (0.8)	1 (0.5)	1 (0.7)	0 (0)	0 (0)	0 (0)
Other^e^	8 (0.8)	1 (0.8)	1 (0.5)	0 (0)	3 (1.2)	3 (1.9)	0 (0)
Primary caregiver marital status^f^							
Single	353 (42.5)	18 (14.3)	77 (39.9)	11 (7.7)	168 (69.7)	1 (10.0)	78 (66.7)
Married	327 (39.4)	58 (46.0)	68 (35.2)	85 (59.4)	68 (28.2)	9 (90.0)	39 (33.3)
Domestic partner	150 (18.1)	50 (39.7)	48 (24.9)	47 (32.9)	5 (2.1)	0 (0)	0 (0)
Primary caregiver age, median (range)	28 (14–66)	29 (16-43)	28 (14–43)	28 (16–47)	28 (14–66)	29 (17–47)	27 (16–42)
Primary caregiver race/ethnicity^g^							
Hispanic/Latinx	407 (52.9)	0 (0)	104 (62.7)	12 (80.0)	120 (58.0)	102 (71.8)	69 (59.5)
White	160 (20.8)	116 (94.3)	5 (3.0)	1 (6.7)	8 (3.9)	30 (21.1)	0 (0)
Black	157 (20.4)	4 (3.3)	45 (27.1)	1 (6.7)	56 (27.1)	4 (2.8)	47 (40.5)
Asian	35 (4.6)	3 (2.4)	6 (3.6)	1 (6.7)	20 (9.7)	5 (3.5)	0 (0)
Pacific Islander	6 (0.8)	0 (0)	4 (2.4)	0 (0)	1 (0.5)	1 (0.7)	0 (0)
Native American	4 (0.5)	0 (0)	2 (1.2)	0 (0)	2 (1.0)	0 (0)	0 (0)
Secondary caregiver^h^							
Father	585 (96.7)	111 (97.4)	145 (99.3)	11 (100)	193 (93.2)	30 (96.8)	95 (99.0)
Grandparent	7 (1.2)	1 (0.9)	0 (0)	0 (0)	6 (2.9)	0 (0)	0 (0)
Mother	7 (1.2)	2 (1.8)	1 (0.7)	0 (0)	3 (1.4)	1 (3.2)	0 (0)
Other caregiver	5 (0.8)	0 (0)	0 (0)	0 (0)	4 (1.9)	0 (0)	1 (1.0)
Legal guardian	1 (0.2)	0 (0)	0 (0)	0 (0)	1 (0.5)	0 (0)	0 (0)
Secondary caregiver age, median (range)	30 (17–59)	32 (19–59)	30 (17–51)	35 (19–55)	30 (17–53)	24.5 (17–36)	31 (19–50)
Number of adults in home^i^							
1	70 (7.4)	7 (5.5)	16 (8.7)	9 (6.5)	16 (6.9)	13 (8.4)	9 (7.8)
2	651 (68.5)	106 (83.5)	101 (55.2)	111 (80.4)	145 (62.5)	108 (70.1)	80 (69.0)
3	130 (13.7)	11 (86.6)	31 (16.9)	9 (6.5)	47 (20.3)	18 (11.7)	14 (12.1)
4 or more	99 (10.4)	3 (2.4)	35 (19.1)	9 (6.5)	24 (10.3)	15 (9.7)	13 (11.2)
Number of children in home^j^							
1	349 (39.3)	48 (37.8)	77 (41.6)	59 (45.7)	69 (30.3)	58 (37.9)	38 (58.5)
2	274 (30.9)	49 (38.6)	56 (30.3)	34 (26.4)	76 (33.3)	43 (28.1)	16 (24.6)
3	164 (16.6)	19 (15.0)	28 (15.1)	23 (17.8)	5 (25.9)	31 (20.3)	4 (6.2)
4 or more	100 (10.1)	11 (8.7)	24 (13.0)	13 (10.1)	24 (10.5)	21 (13.7)	7 (10.8)
Primary language spoken at home^k^							
English	620 (63.4)	124 (97.6)	83 (43.0)	97 (67.8)	196 (80.3)	101 (65.2)	19 (16.4)
Spanish	239 (24.4)	0 (0)	67 (34.7)	46 (32.2)	33 (13.5)	39 (25.2)	54 (46.6)
English & Spanish	34 (3.5)	0 (0)	9 (4.7)	0 (0)	5 (2.0)	13 (8.4)	7 (6.0)
Other^l^	85 (8.7)	3 (2.4)	34 (17.6)	0 (0)	10 (4.1)	2 (1.3)	36 (31.0)

^a^ This site did not reliably collect data on primary caregiver race/ethnicity or the secondary caregiver's relationship to child.

^b^ This site did not reliably collect data on primary marital status or the secondary caregiver's relationship to child.

^c^ There was 1 family with a child of unknown sex.

^d^ There were 4 families with unknown primary caregiver relationship to child.

^e^ 4 Foster parents, 2 Legal guardians, 1 Grandparent, 1 Other caregiver.

^f^ Percentages calculated among 830 families with known primary caregiver marital status.

^g^ Percentages calculated among 769 families with known primary caregiver race.

^h^ Percentages calculated among 605 families with known secondary caregiver relationship to child.

^i^ Percentages calculated among 950 families with known number of adults at home.

^j^ Percentages calculated among 887 families with known number of children at home.

^k^ There were 11 families with unknown primary language spoken at home.

^l^ Amharic, Arabic, ASL, Bengali, Creole, Dari, French, Igbo, Khmer, Mam, Nepali, Pashto, Popti, Portuguese, Punjabi, Russian, Samoan, Sinhala, Swahili, Tagalog, Tamil, Thai, Tigrigna, Turkish, Vietnamese, Yoruba, English & Other, Spanish & Other.

**Table 3:** Demographics of the early childhood system's county catchment area, DULCE families, and DULCE teams. .

Demographics	Appleseed –Lamoille County, VT			Highland –Alameda County, CA			Los Angeles County, CA						Brumback – Palm Beach County, FL		
							Northeast Valley		Newhall		Children's Clinic^a^				
Social Vulnerability Index^b^	0.131			0.555			0.869		0.869		0.869		0.796		
	County	DULCE	Team	County	DULCE	Team	County^c^	DULCE	DULCE	Team^d^	DULCE	Team	County	DULCE	Team
Total (N)^e^	346	127	7	25,809	194	6	76,648	146	155	11	249	9	21,014	118	11
Race/Ethnicity^f^ (%)															
Hispanic/Latinx	2.0%	0%	0%	22.4%	62.7%	50%	49.1%	80.0%	71.8%	63.7%	58.0%	44.4%	23.9%	59.5%	27.3%
White	94.2%	94.3%	85.7%	29.2%	3.0%	33.3%	25.3%	6.7%	21.1%	18.2%	3.9%	44.4%	52.6%	0%	36.4%
Black	1.2%	3.3%	0%	10.7%	27.1%	16.7%	9.0%	6.7%	2.8%	9.1%	27.1%	0%	20.1%	40.5%	36.4%
Asian	0.7%	2.4%	0%	33.8%	3.6%	0%	15.6%	6.7%	3.5%	9.1%	9.7%	11.1%	3.0%	0%	0%
Pacific Islander	0.1%	0%	0%	1.0%	2.4%	0%	0.4%	0%	0.7%	0%	0.5%	0%	0.1%	0%	0%
Native American	0.4%	0%	0%	1.1%	1.2%	0%	1.5%	0%	0%	0%	1.0%	0%	0.6%	0%	0%
Multiracial	1.8%	0%	0%	5.6%	N/A	N/A	3.3%	N/A	N/A	N/A	N/A	0%	1.9%	N/A	N/A
Not reported	N/A	N/A	14.3%	N/A	N/A	N/A	N/A	N/A	N/A	N/A	N/A	N/A	N/A	N/A	N/A
Language other than English spoken^f^ (%)	3.5%	2.4%	14.3%	46.0%	57.0%	66.7%	55.8%	32.2%	34.8%	72.7%	19.7%	66.7%	33.1%	83.6%	36.4%
Average N household members^f^	2.33	4.04	N/A	2.82	4.53	N/A	2.94	4.08	4.36	N/A	4.53	N/A	2.51	3.96	N/A

N/A; data not available or not collected.

^a^ The Children's Clinic includes data for 2 DULCE clinics: Central Long Beach (established site) and S. Mark Taper Foundation (new site).

^b^ The Social Vulnerability Index uses U.S. Census data to determine the relative level of social vulnerability of each county: https://www.atsdr.cdc.gov/placeandhealth/svi/interactive_map.html, 0.131 = low, 0.555 = medium to high, 0.796 and 0.869 = high.

^c^ The county-level data for Los Angeles County applies to all three sites: Northeast Valley, Newhall, Children's Clinic.

^d^ Northeast Valley and Newhall shared the same DULCE team.

^e^ County totals represent the newborn population in each county over the study period.

^f^ County-level data from 2020 U.S. census. These demographic data are not restricted to families with infants: https://www.census.gov/quickfacts/fact/table/US/PST045221.

**Table 4:** DULCE's reach within each site's pediatric clinic(s) and affiliated healthcare system.

Healthcare System	Appleseed – Lamoille County, VT	Highland – Alameda County, CA	Los Angeles County, CA			Brumback^a^ – Palm Beach County, FL
			Northeast Valley	Newhall	Children's Clinic	
	N (%)	N (%)	N (%)	N (%)	N (%)	N (%)
Pediatric clinics in system	3 (100)	5 (100)	9 (100)		9 (100)	2 (100)
Pediatric clinics that implemented DULCE	1 (33.3)	1 (20.0)	2 (22.2)		2 (22.2)	1 (50.0)
Infants that received care at all clinics in the system	259 (100)	1,558 (100)	3,632 (100)		2,182 (100)	26,316 visits
Infants that enrolled in DULCE among all infants receiving care in system	127 (49.0)	194 (12.5)	301 (8.3)		249 (11.4)	118 (n/a)
Infants that received care at DULCE-implementing clinic(s)	142 (54.8)	388 (24.9)	580 (16.0)		1,741 (79.8)	10,574 visits
Infants that enrolled in DULCE among all infants at DULCE-implementing clinics	127 (89.4)	194 (50.0)	146 (25.2)	155 (26.7)	249 (14.3)	118 (n/a)

^a^ The C.L. Brumback Primary Care site provided the number of pediatric visits over the study period, not the number of infants served.

Table 3 also includes the Social Vulnerability Index (50) for each county: Lamoille County has low vulnerability, Alameda County has medium to high vulnerability, while Los Angeles and Palm Beach counties have a high level of vulnerability. In these latter three counties, Hispanic/Latinx and Black families are overrepresented among DULCE families, relative to county-level demographics. This overrepresentation reflects the intention to launch DULCE in clinics with high Medicaid-enrolled populations, which tend to have higher proportions of Hispanic/Latinx and Black patients.

### 2.5. Analysis

To answer the first research question – can a lighter-touch application of BTS sustain improvements achieved during a prior expansion by four established DULCE teams and spread improvements in on-time WCVs and identification and support for HRSN to three new clinics? – we first calculated descriptive statistics for measures of implementation fidelity for all sites together and for each clinic-based team separately: DULCE enrollment and completion rates, number of weeks enrolled, total number of encounters per family, percent of families discussed in case review at least once, and FS-family contact time. We compared these values to benchmark values from phase one, except for the case review measure which was collected for the first time during phase two.

To determine if improvements were sustained and spread, we calculated the process measures associated with DULCE's primary drivers and the outcome measure associated with its main aim for all sites together and for each clinic-based team separately. We then analyzed process and outcomes in time series as run charts, well-established methods that can identify changes that are unlikely due to chance alone and allow inferences to be drawn from the temporal relationships of interventions and results (51). Subjects were counted in the denominator of each measure once (i.e., denominators are independent of each other) and placed in the month they enrolled (all WCVs on time; all HRSN measures), in the month the data point occurred (FS WCV attendance), or, for the 1-month WCV timeliness measure, in the month containing the last day of the 1-month visit window.

Means were used because these were non-continuous data, and some measures' medians were extreme values due to high baseline performance (e.g., baseline median screening rates of 100%) and small site-level denominators (e.g., months with few or no identified IPV cases). Criteria for applying probability-based rules for identifying improvements were met: denominators were roughly equal over time, and at the aggregate level, data was appropriately dispersed (52).

Two probability-based rules were used to identify changes in the data that have less than 5% probability of occurring by chance: a “shift” of six or more points in a row above or below the mean, and a “trend” of five consecutive increasing or decreasing points (53). When a shift occurred, the average of the six shifted points became the new mean, from which subsequent shifts were identified.

To answer the second research question – what proportion of families use resources after receiving resource information for identified HRSN? – for families that screened positive for HRSN, we calculated the proportion of families that were provided resource information and the proportion that used HRSN resources for caregiver depression and/or IPV, for concrete supports, and for each HRSN separately.

Analyses were conducted using Stata 14.2.

### 2.6. Ethical considerations

The University of Chicago School of Social Administration's Institutional Review Board (IRB17-0414) approved this study.

## 3. Results

Table 2 describes the analytic sample. Families of 989 infants born June 1, 2018 through December 31, 2019 participated; 98.9% of primary caregivers were mothers whose median age was 28 years. Forty-three percent were single; 20.8% identified as White, 20.4% as Black, and 52.9% as Hispanic/Latinx. Sixty-one percent of families reported a second caregiver: 96.7% were fathers, whose median age was 30 years. Families mainly spoke English (63.4%) or Spanish (24.4%). Families represented the demographics of the clinics' populations and were similar to families in DULCE's phase one expansion (44). The level to which families reflected county-level demographics (i.e., the early childhood system's catchment area) varied by site (Table 3). For example, the demographic characteristics of DULCE families at Appleseed Pediatrics were similar to those of Lamoille County, but Highland Hospital in Oakland, CA (a safety-net hospital) served a much higher proportion of Hispanic/Latinx and Black families than are represented in Alameda County's demographic data.

To answer the first research question – can a lighter-touch application of BTS sustain improvements achieved during a prior expansion by four established DULCE teams and spread improvements in on-time WCVs and identification and support for HRSN to three new clinics? – we first consider the main outcome (i.e., on-time WCVs) then implementation and process measures. We compare them to the phase one outcome, implementation, and process measures.

**Figure 5:**
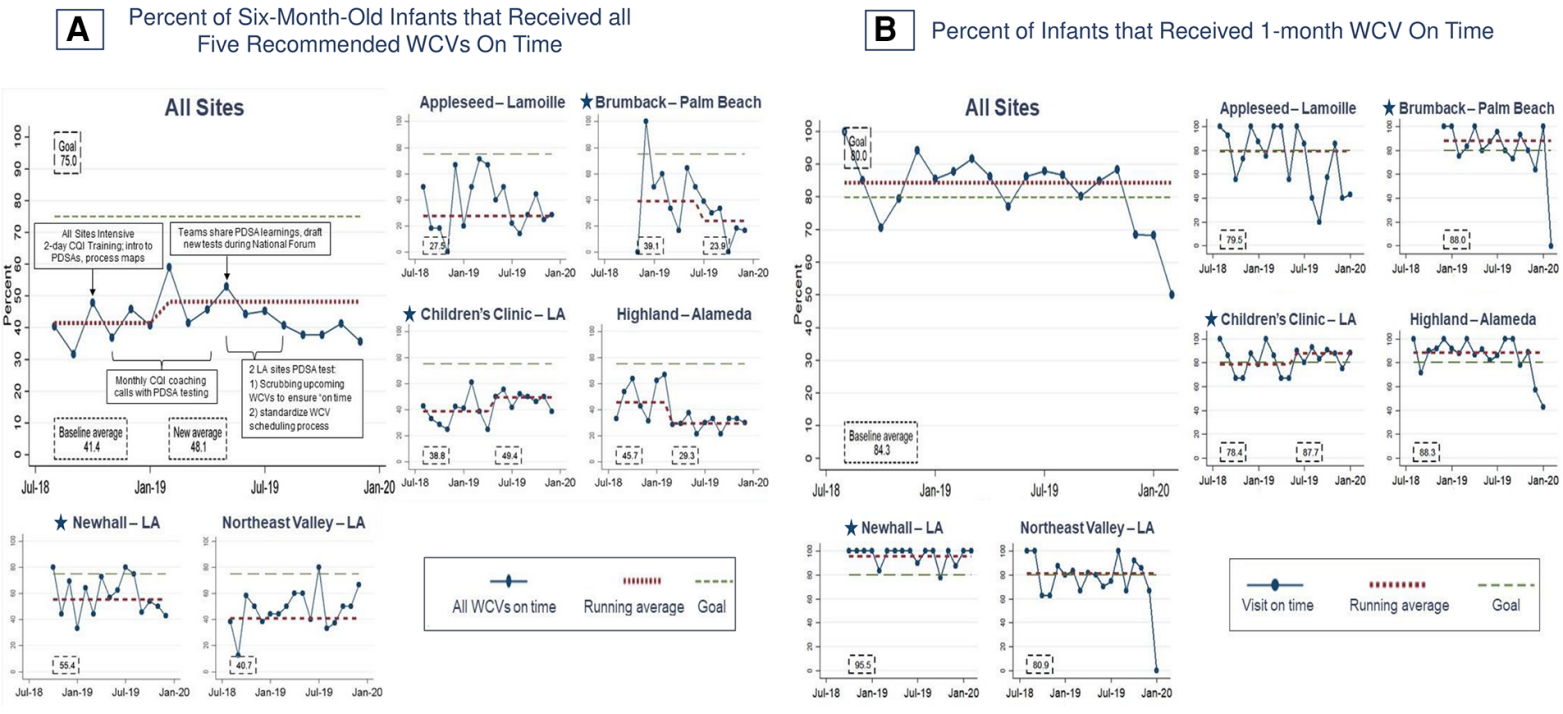
Percent of infants that received recommended well-child visits on time. Star symbols denote new DUCLE sites: Brumback (Palm Beach County), The Children's Clinic [Los Angeles (LA) County], which aggregated data for its established and new DULCE clinics, and Newhall, part of the Northeast Valley Health Corporation (LA county), a previously established DULCE site.

Figure 5 shows the main outcome: at baseline, 41.4% of six-month-old infants completed all five recommended WCVs on time. A shift to 48.1% occurred at six months (February 2019). One clinic (Children's Clinic–LA) demonstrated an increase from 38.8% to 49.4% that correlated with improved 1-month WCVs timeliness (78.4% to 87.7%) when they tested standardized scheduling at their two clinics (one established and one new). Overall, this is comparable with phase one, during which sites demonstrated a shift from a baseline average of 45.8% to 65.4% that also was associated with improvements in on-time 1-month WCVs at three sites (Appleseed–Lamoille, Highland–Alameda, and Northeast Valley–LA). The more modest improvement might be explained, at least in part, by the decrease in preventive care by infants and their families during the COVID-19, which affected this measure for all infants born after August 2019. In addition, during phase two, one established site and one new site experienced downward shifts concurrent with the transition from individual site-level monthly coaching with data provided by DULCE National to quarterly Learning Network Calls and local data management: from 45.7% to 29.3% at Highland–Alameda (March 2019), and 39.1% to 23.9% at Brumback–Palm Beach (July 2019).

Overall, implementation measures were similar between phases one and two. All families offered DULCE enrolled, and 73.8% completed the six-month intervention, mirroring enrollment and completion rates observed in phase one (100% and 79%, respectively). Nearly two-thirds of families that left early moved away or changed clinics (Table 5), like in phase one. In phase two, enrolled families had a median intervention dose of 26 weeks [Interquartile Range (IQR), 20–28], nine encounters (IQR, 6–14), and 180 min of FS contact time (IQR, 120–295), compared to 24 weeks [confidence interval (CI), 23.1–24.2], 11 encounters (CI, 10.3–11.1), and 280 min (CI, 265–294) in phase one. During phase two, teams discussed 67% of families in case review within two months of DULCE enrollment; this measure was not collected in phase one.

**Table 5:** DULCE enrollment, completion and reasons for leaving early.

	Total	Appleseed – Lamoille County, VT	Highland – Alameda County, CA	Northeast Valley – LA County, CA	Children's Clinic – LA County, CA	Newhall – LA County, CA	Brumback – Palm Beach County, FL
	*N* (%)	*N* (%)	*N* (%)	*N* (%)	*N* (%)	*N* (%)	*N* (%)
Families offered DULCE	989	127	194	146	249	155	118
Families enrolled in DULCE	989 (100)	127 (100)	194 (100)	146 (100)	249 (100)	155 (100)	118 (100)
Families that completed DULCE	730 (73.8)	113 (89.0)	141 (72.7)	87 (59.6)	186 (74.7)	117 (75.5)	86 (72.9)
Families that left DULCE early^a^	259 (26.2)	14 (11.0)	53 (27.3)	59 (40.4)	63 (25.3)	38 (24.5)	32 (27.1)
Reasons for leaving early^a^							
Moved home	101 (39.0)	10 (71.4)	12 (22.6)	25 (42.4)	35 (55.6)	15 (39.5)	4 (12.5)
Change clinic or provider	58 (22.4)	2 (14.3)	15 (28.3)	0 (0)	15 (23.8)	11 (28.9)	15 (46.9)
Lost to follow-up	41 (15.8)	2 (14.3)	19 (35.8)	0 (0)	3 (4.8)	7 (18.4)	10 (31.3)
Family requested	17 (6.6)	0 (0)	4 (7.5)	2 (3.4)	4 (6.3)	5 (13.2)	2 (6.3)
Baby died or removed from home	2 (0.8)	0 (0)	1 (1.9)	0 (0)	1 (1.1)	0 (0)	0 (0)
Other	5 (1.9)	0 (0)	2 (3.8)	0 (0)	2 (3.2)	0 (0)	1 (3.1)
Missing	35 (13.5)	0 (0)	0 (0)	32 (54.2)	3 (4.8)	0 (0)	0 (0)

^a^ Families that left DULCE prior to completing their six-month well-child visit.

**Figure 6:**
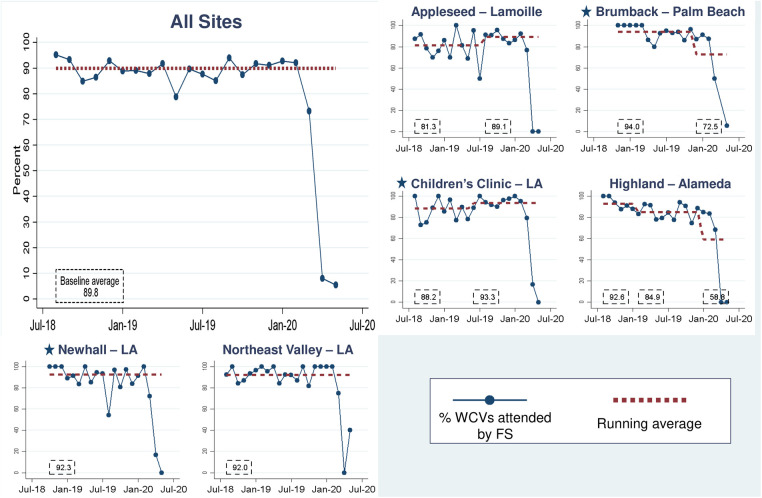
Percent of well-child visits (WCVs) attended by the family specialist. Star symbols denote new DUCLE sites: Brumback (Palm Beach County), The Children's Clinic [Los Angeles (LA) County], which aggregated data for its established and new DULCE clinics, and Newhall, part of the Northeast Valley Health Corporation (LA county), a previously established DULCE site.

Figure 6 shows that the process measure for FS present in WCVs (PD1) was high at baseline and remained stably high throughout phase two: in aggregate, FS attended 89.8% of WCVs (Figure 6). This exceeded phase one performance, which improved from 0% to 66% to 70%. Two phase two new sites immediately achieved and sustained high FS presence: Newhall–LA (92.3%) and Brumback–Palm Beach (92.0%). At the Children's Clinic–LA site, which reported data for its established site and new site together, FS presence in WCVs dipped to 88.2% when the new SMTF site joined, then shifted to 93.3% in October 2018, coincident with the two-day, in-person onboarding. All sites demonstrate a sharp decline in the first quarter of 2020 that corresponds to clinic safety protocol changes in response to the COVID-19 pandemic (Figure 6).

**Figure 7:**
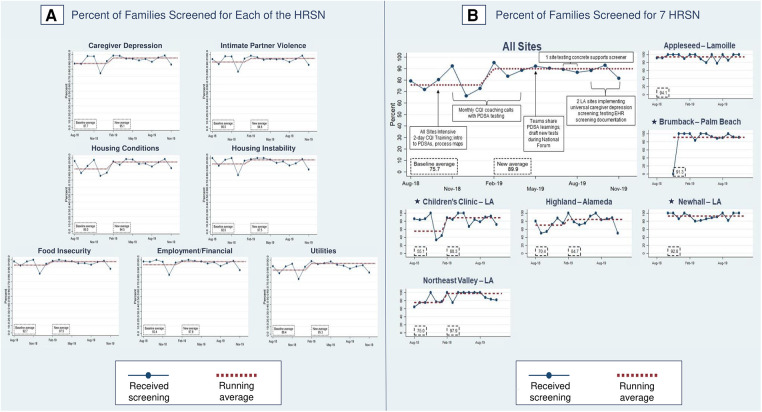
Percent of families screened for health-related social needs (HRSN). Star symbols denote new DUCLE sites: Brumback (Palm Beach County), The Children's Clinic [Los Angeles (LA) County], which aggregated data for its established and new DULCE clinics, and Newhall, part of the Northeast Valley Health Corporation (LA county), a previously established DULCE site.

Figure 7 shows process measures for HRSN screening (PD2). The phase two aggregate measure of families screened for all seven HRSN improved from a baseline of 75.7% to 89.9%, with three sites demonstrating shifts: one mixed new-established site [55.1% to 88.3% (Children's Clinic–LA)] and two established sites [70.4% to 84.7% (Highland–Alameda), 75.0% to 97.9% (Northeast Valley–LA)]. In phase one, sites screened 92% of families for all seven HRSN.

For each HRSN, screening rates in phase two were 85.0%–93.4% at baseline and improved, with shifts at 6 months in caregiver depression (87.7% to 95.1%), IPV (90.0% to 94.8%), housing conditions (85.0% to 94.5%), housing instability (92.0% to 97.5%), food insecurity (92.7% to 97.5%), employment/financial needs (93.4% to 97.8%), and utilities (86.4% to 95.3%). Improved rates were similar with phase one screening performance: caregiver depression (95.9%), IPV (96.3%), housing conditions (shift from 94.5% to 95.8%), housing instability (97.2%), food insecurity (97.2%), employment/financial needs (98.6%), and utilities (96.8%).

**Figure 8:**
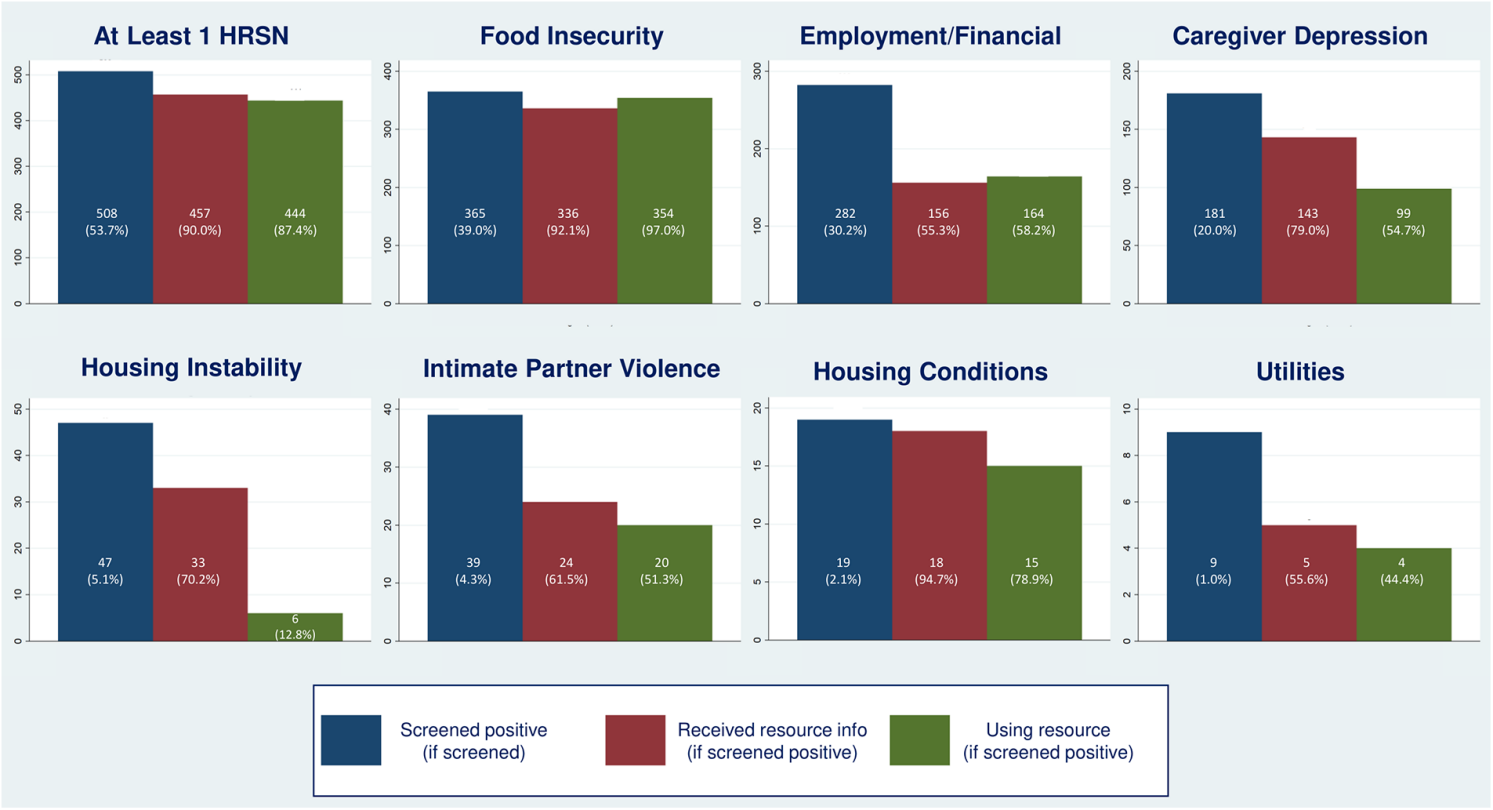
Identification and support for DULCE families’ health-related social needs (HRSN), by HRSN domain.

In phase two, 54% of families had at least one positive HRSN screen: 20% had two and 11% had three or more. This differed from phase one, where 70% of families had at least one positive screen: 25% had two and 16% had three or more. Prevalence of individual HRSN varied as well between phase two and phase one: for food insecurity, 39.0% of phase two families vs. 46.1% of families in phase one; for employment/financial needs, 30.2% vs. 51.0%; for caregiver depression, 20% vs. 14.3%; for housing instability, 5.1% vs. 13.2%; for IPV, 4.3% vs. 5.1%; for unhealthy housing conditions, 2.1% vs. 3.5%, and for utility needs, 1.0% vs. 2.2% (Figure 8).

**Figure 9:**
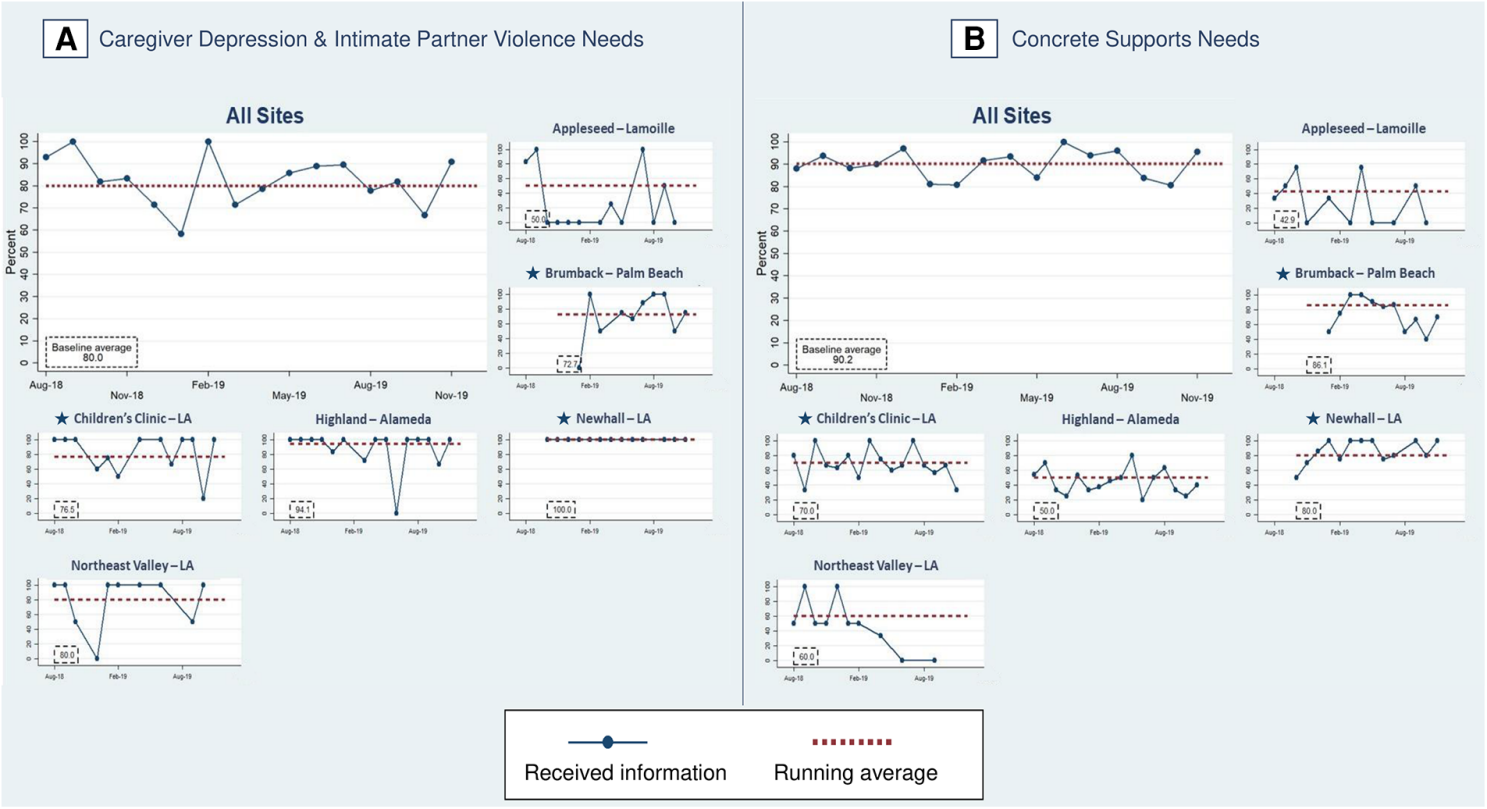
Percent of families with identified health-related social needs that received information about available resources. Star symbols denote new DUCLE sites: Brumback (Palm Beach County), The Children's Clinic [Los Angeles (LA) County], which aggregated data for its established and new DULCE clinics, and Newhall, part of the Northeast Valley Health Corporation (LA county), a previously established DULCE site.

In phase two, 80% of families experiencing caregiver depression and/or IPV and 90% of families with concrete supports needs received resource information (PD2) (Figure 9), an increase from 70.7% of families with caregiver depression and/or IPV and 86.4% with concrete support needs at the end of phase one. In phase two, FS provided resource information to 92.1% of families with food insecurity, 55.3% with employment/financial needs, to 79.0% of depressed caregivers, 70.2% with housing instability, 61.5% of families with IPV, 94.7% with unhealthy housing conditions, and 55.6% with utility needs (Figure 8).

**Figure 10:**
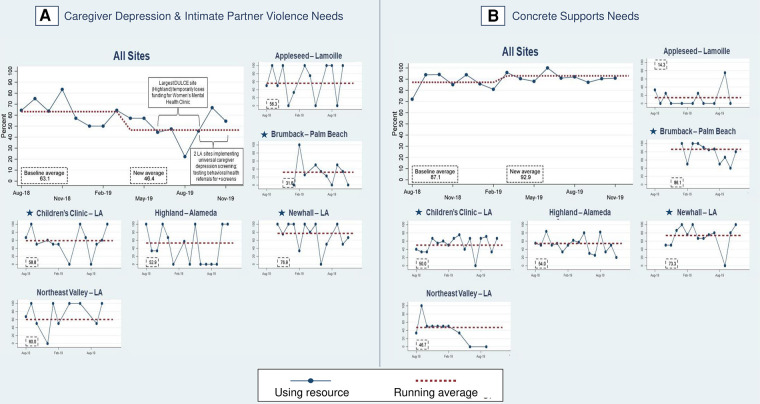
Percent of families with identified health-related social needs using resources. Star symbols denote new DUCLE sites: Brumback (Palm Beach County), The Children's Clinic [Los Angeles (LA) County], which aggregated data for its established and new DULCE clinics, and Newhall, part of the Northeast Valley Health Corporation (LA county), a previously established DULCE site.

The last process measure – the percent of families with HRSN that used resources (PD3) – responds to this study's second research question with new data that was not collected in phase one. Among families with at least one identified HRSN, 87.4% successfully used at least one related resource (Figure 8). The percent of families that used concrete supports resources increased from 87.1% to 92.9% of families (Figure 10). Resource use varied by HRSN (Figure 8): food insecurity (97.0%), employment/financial needs (58.2%), caregiver depression (54.7%), housing instability (12.8%), IPV (51.3%), housing conditions (78.9%), and utilities (44.4%).

## 4. Discussion

This study examined the results of scaling a pediatric clinic-based EBI using a lighter-touch CQI approach. Scaling within existing healthcare and early childhood systems capitalized on existing relationships, infrastructure, and experience, allowing for lighter-touch CQI support that reduced implementation and maintenance costs. The main outcome (percent of infants who received all recommended WCVs on time) initially dropped to a similar baseline average (41.4%) seen in the first phase expansion, then demonstrated modest improvement to 48.1%, despite the lower use of preventive care during the initial phase of the COVID-19 pandemic (54).

Other process measures concerning families’ WCVs (1-month WCV timeliness, FS presence) and HRSN (rates of screening, resource discussion, and – newly – resource use) were maintained or improved. These results shed insight into the varying levels of CQI support needed to introduce or sustain different intervention components.

Many improvements from DULCE's phase one expansion spread quickly to new sites and further improved at established sites. DULCE practice change, namely FS presence in WCVs, increased from 0 to 66% over 10 months of intensive support during phase one, then reached 89.8% in the first six months of this second phase (until COVID-19 necessitated limits to in-person contact). The second phase benefitted from innovations in phase one, including providers acceptance of FS presence and CQI-driven changes in scheduling practices and clinic workflows (44). This measure's continued improvement suggests that once FSs are integrated into clinical teams, they become an essential, sustainable part of clinic function.

Similarly, HRSN screening and resource discussion processes were sustained by established sites and spread to new sites with lighter-touch CQI support. As is commonly observed in efforts to disseminate EBIs (8, 46, 52), integration of new sites at the beginning of phase two produced a dip in performance that recovered quickly – i.e., phase two's aggregate baseline screening rates were slightly lower than late phase-one levels, but reached very high rates within six months. For example, one site (The Children's Clinic–LA) took one year to improve screening for seven HRSN from 92% to 100% of families in phase one, then dropped to 50% when a second, much larger site (SMTF) was added, and recovered to 88% within six months.

Accelerated adoption by new sites and ongoing improvement of established sites reflects intentional design that included all actors in phase one, then drew on them to harvest tested implementation strategies and leverage the power of peer champions to introduce innovations (as described in the literature) (8, 14, 55, 56). In this case, two of the three new clinic sites were within the same healthcare delivery system as established sites, and clinical leadership benefitted from integrating into their “new” DULCE site teams the same legal partners and early childhood system leaders as the established sites. Such design allows for tapering the intensity of support while maintaining early adopters' outcomes and spreading improvements to additional sites (57).

However, not all phase-one improvements were sustained. The main outcome regressed to pre-phase one levels (∼41% of six-month-old infants received all WCVs on time at baseline in both phases) then improved to 48.1% but did not approach the 66% achieved during phase one. Those infants born after September 2019 may have missed visits due to pandemic-related disruptions; follow-on studies would determine whether performance improved as disruptions eased. In addition, none of the DULCE sites were able to monitor this measure during phase two, preventing data-driven CQI activities.

Most of phase one's improvement was associated with improvements in the 1-month WCV, fueled by learning that Medicaid covered this newly recommended visit (44, 58). In phase one, on-time 1-month WCVs improved from 62.5% to 79.5%. The healthcare systems spread the learning across all their sites – including the new DULCE sites – so that by the beginning of phase two, 84.3% of 1-month WCVs were on time. Like FS presence and HRSN process measures above, DULCE teams sustained and spread phase-one improvements in 1-month WCVs.

HRSN screening intends to identify and address family needs. In this second-phase study, we collected data on families’ HRSN resource use – addressing a limitation of the many studies that measure referrals but not whether families successfully use the resources (59–62). Connection rates varied by domain (13%–97%), and likely reflect clinics' relationships with different service providers and systemic barriers. Connection rates were highest for food (97%), housing conditions (79%), and employment/financial needs (58%). Over half of families with caregiver depression (55%), and IPV (51%) used resources, compared to four of nine families with utility needs (44%). Use of housing instability resources was lowest (13%), reflective of the affordable housing crisis.

These are promising results. Clinic-based HRSN interventions often struggle to link patients to resources: a CQI collaborative in 19 pediatric clinics increased HRSN screening from 19% to 73%, but did not increase HRSN referrals (63). A recent systematic review suggested that direct referrals or additional assistance with indirect referrals improved patient outcomes compared with indirect referrals only (64). Other pediatric clinic-based interventions that collected data on resource use demonstrated a wide range of connection rates (0.8%–75%), and most EBIs only address a single HRSN domain (61, 65–73).

Families with multiple HRSN often must navigate multiple programs that address single domains of need (74). Breaking down silos between healthcare, public health, legal, and early childhood systems serves families more equitably and efficiently. DULCE's FS and cross-sector team is designed to support families to navigate resources in a streamlined, cohesive manner, often utilizing warm handoffs to facilitate connections. This level of care coordination and family-led problem solving is not feasible for clinicians alone to execute; team-based, cross-sector care distributes this responsibility and strengthens relationships with families. The weekly cross-sector team meetings supported maintenance of this collaborative teamwork at each site.

Continuous quality improvement methods, including BTS, are designed to improve the original intervention and simultaneously promote sustainability in scale-up. Successful CQI efforts develop strong improvement teams that involve stakeholders, institutional leaders, frontline service providers, as well as patients; intentionally develop infrastructure and organizational capacity to support practice changes and a culture of CQI; and rely upon iterative use of data and feedback loops to inform practice changes. It flattens the hierarchy of decision-making within teams and balances power, elevating the voices of those who typically do not contribute to leadership decisions but whose perspectives are invaluable (e.g., frontline workers, families).

Continuous quality improvement also builds teams' capacities to solve their own problems and use data to identify areas of improvement. It transforms the way data is typically used (i.e., from a judgement to a learning opportunity). Furthermore, PDSAs require teams to start testing small (e.g., 1 patient interaction), empowering them to initiate rapid-cycle testing and learn from failure. While many implementation frameworks stress fidelity of implementation, CQI focuses on local adaptation informed by real-time data to retain fidelity to process and outcome measures. These cross-hierarchical, inquiry-driven tools strengthen not just the implementation of one EBI – in this case, DULCE – but these experiences build teams' capacity for learning through iterative trial-and-error, which benefits the entire clinical ecosystem, including parallel social determinants of health (SDOH) efforts and patients not enrolled in any interventions.

While offering many benefits to clinical teams and healthcare systems, CQI collaboratives such as BTS are resource intensive. Many scale-up frameworks intentionally design an early phase of expansion with intensive support to a core set of participants that represent all actors and relationships in a system. For example, McCannon et al. (46) and Barker et al. (8) both describe large-scale spread of EBIs that intentionally selected a small “wedge” or “scalable unit” — a microcosm of the entire system — to begin. By providing intensive support at a smaller scale, these efforts learn not only what needs to be adapted as the EBI spreads, but how to support teams in their adaptation (i.e., what infrastructure is needed). After this initial intensive learning phase, subsequent scale phase(s) is driven by activating induction-phase participants as peer mentors to help spread the EBI to additional “wedges.” (14, 56) In these subsequent phases, improvement is often accelerated – as it was in this DULCE expansion.

With respect to clinics' CQI-driven learnings, several themes emerged. First, identifying HRSN is not risk-free for families (75), particularly those with immigration issues during this study period when the Trump administration's public charge rule was in effect (76). FS built rapport and trust with families over their six-month enrollment, allowing families time to disclose sensitive or stigmatized needs (which typically occurred around the 4-month WCV), and were supported with accurate legal information from the legal partner at each site.

Family-centered care includes respecting families' desires around their HRSN. For example, some parents with infants tolerated overcrowded housing conditions rather than lose their proximity to extended family supports. Better understanding family agency amd priorities is a future area of research within DULCE. In such complex cases, the cross-sector team's diverse expertise generates creative problem solving that adapts solutions to families' circumstances and preferences. Cultivating a consistent, trusting relationship between the DULCE family and their entire care team makes family-led problem solving possible.

Continuous quality improvement also allows sites to develop culturally-aware approaches to some challenges. In some communities, stigma discourages parents from seeking support for postpartum depression. At two sites, the DULCE team collaborated to sidestep this stigma through careful messaging and the use of more acceptable resources. One clinic referred to a Fussy Baby clinic that included parent-child psychotherapy, and another one developed an infant massage class led by a mental health provider. Differences in local culture, resources, and team resourcefulness may have contributed to this cross-site variability.

This study contributes to the literature on CQI strategies for scaling and sustaining healthcare-based EBIs to improve population health. It also reports the results of families' rates of HRSN resource use, an outcomes-oriented indicator that is often omitted in SDOH interventions.

### 4.1. Limitations

The selection of volunteer sites for implementing DULCE limits generalizability. Our analyses identify improvements that are unlikely due to chance alone but lack causal inference. External events, notably the COVID-19 pandemic, may have affected some measures of reach and effectiveness. This study relied on data reported by Family Specialists for CQI purposes which did not include balancing measures; ongoing work will incorporate data about contextual factors and stakeholders' perspectives, E.H.R and claims data for participants and a comparison group (77).

## 5. Conclusion

An innovative, lighter-touch CQI approach to a second phase of scale-up resulted in maintenance or improvements in most processes and outcomes at four established clinics and three new clinics. Outcomes-oriented CQI measures (family HRSN resource use) are an important addition to more traditional process-oriented indicators.

## Data Availability

The raw data supporting the conclusions of this article will be made available by the authors, without undue reservation.
