# Supplementary material for: Sustaining and scaling a clinic-based approach to address health-related social needs
Source: Front Health Serv. 2023 Feb 17;3:1040992. doi: 10.3389/frhs.2023.1040992 (PMC10012656; doi:10.3389/frhs.2023.1040992)
Supplement: Supplementary file 1 [file Supplement1.docx]

| **PDSA Worksheet**  **Team Name:** | |
| --- | --- |
| Addressing Primary Driver # _____  Cycle #______ | Start Date: End Date: |
| Objective of Cycle  ___ Test a Change  ___ Implement change | **Please Describe:**  What are you trying to accomplish?  How will you know a change is an improvement?  What ideas do you have that will result in an improvement? |
| Tasks needed to complete test (check all that apply) | 🞏 Collect data to inform change by __________  🞏 Talk with or educate staff by ______________  🞏 Discuss test with leadership by ____________  🞏 Review test with another department by __________  🞏 Other (describe) |
| PLAN (P) | **Question you want answered with this test**  If we:  Will it result in:  **Plan for this test**   1. What: 2. Who: 3. When: 4. Where: 5. Why:­   **Plan for collection of data** (Depends on what question test aims to address)   1. What data will be collected? 2. How will data be collected? 3. When will the data be collected?   **Predict what will happen as a result of this test**  Prediction:  If your prediction is on target, what might your next test of change be? |
| **STOP here: Teams will not be able to complete the Do-Study-Act portion until you run the test.** | |
| (D) | **Observations**  Was the test carried out as planned? Did you modify from the original plan? If so, how?  What did you observe that wasn’t part of the plan?  Begin analysis of data (graph data, picture, etc.) – *example below:*   \| Week of \| N newborns w WCV \| N given DULCE postcard \| N engaged in follow-up call \| \| --- \| --- \| --- \| --- \| \|  \|  \|  \|  \| \|  \|  \|  \|  \| \|  \|  \|  \|  \| \|  \|  \|  \|  \| \|  \|  \|  \|  \| |
| STUDY (S) | **Complete analysis of the data**  Did your results match your predictions?  What did you learn?  What did your data tell you? |
| ACT (A)  ___Adapt*  ___Adopt**  ___Abandon*** | Describe next PDSA cycle  New Questions to Answer:  **If we**__________________________________________________________________ **will it result in**___________________________________________________________  ________________________________________________________________________________________________________________________________________  Decisions made/Action to take:  ________________________________________________________________________________________________________________________________________ |

*Adapt (Improve plan, continue testing.)

**Adopt (Select changes to implement on larger scale, develop implementation plan, plan for sustainability.)

***Abandon (Test something different.)
